# Preparation and Characterization of Silica Aerogel Microspheres

**DOI:** 10.3390/ma10040435

**Published:** 2017-04-20

**Authors:** Qifeng Chen, Hui Wang, Luyi Sun

**Affiliations:** 1 College of Materials Science & Engineering, South China University of Technology, Guangzhou 510640, China; chenqifeng1125@126.com; 2 Department of Chemical & Biomolecular Engineering and Polymer Program, Institute of Materials Science, University of Connecticut, Storrs, CT 06269, USA

**Keywords:** silica aerogel microspheres, emulsion method

## Abstract

Silica aerogel microspheres based on alkali silica sol were synthesized using the emulsion method. The experimental results revealed that the silica aerogel microspheres (4–20 µm in diameter) were mesoporous solids with an average pore diameter ranging from 6 to 35 nm. The tapping densities and specific surface areas of the aerogel microspheres are in the range of 0.112–0.287 g/cm^3^ and 207.5–660.6 m^2^/g, respectively. The diameter of the silica aerogel microspheres could be tailored by varying the processing conditions including agitation rate, water/oil ratio, mass ratio of Span 80: Tween 80, and emulsifier concentration. The effects of these parameters on the morphology and textural properties of the synthesized silica aerogel microspheres were systematically investigated. Such silica aerogel microspheres can be used to prepare large-scale silica aerogels at an ambient pressure for applications in separation and high efficiency catalysis, which requires features of high porosity and easy fill and recovery.

## 1. Introduction

Aerogels are highly porous and possess remarkable properties such as high specific surface area and low thermal conductivity [1,2,3]. These features render aerogels a promising candidate for widespread application [4,5,6,7], including thermal super-insulators [8], supports for catalysts [9], and drug delivery carriers [7]. For example, Rajanna et al. prepared a high loading drug carrier (0.47 g ibuprofen/g hollow silica aerogel) [10], and Kim et al. synthesized Ni-alumina aerogel catalysts which exhibited a high catalytic activity [11].

Silica aerogels are usually prepared via a sol-gel process and typically use expensive precursors, such as tetraethyl orthosilicate (TEOS) and tetramethyloxysilane (TMOS), through supercritical extraction of pore liquids from the wet gels, meanwhile maintaining their structural integrity and high porosity [12]. However, the complex process and high pressure involved in supercritical drying of gels severely restricts the scaling up of the production of silica aerogels [13]. A method to synthesize silica aerogels using low cost precursors at ambient temperature and pressure is highly desirable. Ambient pressure drying (APD) has proven to be attractive and meet the above requirements. In ambient pressure drying, solvents with a low surface tension are used to replace the solvent in the gel network. Meanwhile, organic groups replace the hydroxyl groups, leading to a reduction of shrinkage of the gel. Sharad et al. used water glass as a precursor and trimethyl chlorosilane (TMCS) and hexamethyldisilazane (HMDS) as surface modifying agents to synthesize aerogel beads (1–2 cm in diameter) by the co-precursor method [14]. Wang et al. reported the synthesis of silica aerogel by APD with ternary azeotropes (H_2_O/n-butanol/n-hexane) as components of pore fluid [15]. However, solvent exchange is a tedious and time-consuming route because of the large dimensions of silica aerogels. Using a huge amount of low surface tension solvents (such as n-hexane) and surface modifying agents (such as TMCS, HMDS) are highly undesirable for our environment and health. Meanwhile, solvent exchange and surface modification are essential for preserving the three-dimensional porous structures of the silica aerogels before APD. Solvent exchange depends on two main factors: surface tension of the solvent and dimensions of the gel [16]. Moreover, centimeter-sized monolithic hydrogels are typically weaker and thus tend to crack during solvent exchange. In the drying process, the lateral compressive stress in the tiny pores on the gel network will increase and the surface silanol groups on the adjacent silica clusters undergo condensation reactions, resulting in irreversible cracks in the gel network [14]. Therefore, the synthesis of large scale silica aerogels at an ambient pressure is particularly challenging. 

A potential approach to address the above challenge is to pre-synthesize silica aerogel microspheres. In this way, the solvent exchange step could proceed quickly (minimal diffusion obstacles), thus allowing a dramatic reduction in synthesis time [17]. Huang et al. reported a silica aerogel glazing system by filling granular silica aerogel in between two layers of glass plates, and the results showed that the system processed excellent thermal insulation [18]. Liu et al. demonstrated the synthesis of titania-silica microspheres by a water-in-oil emulsion technique combined with APD [19]. They used Span 80 and Tween 85 as surfactants, and the hydrophile-lipophile balance (HLB) value of the emulsion was 4.9. It is easy to form a stable water-in-oil emulsion with an increase of the lipophilicity and a decrease of the HLB value of the emulsion [20]. The HLB value of a stabilized water-in-oil emulsion is generally below 6 [20]. However, how the process conditions would affect the dimensions of the synthesized aerogel microspheres is yet to be revealed. Many parameters may influence the size of the synthesized aerogel microspheres, including sol-oil ratio, composite emulsifier composition, and agitation induced shear stress. Precisely controlling the dimension of silica aerogel microspheres will significantly facilitate the fabrication of large scale silica aerogels at an ambient pressure at the next step.

In this present work, we report the synthesis of micrometer-sized (4–20 µm) silica aerogel microspheres at ambient pressure. The processing-silica aerogel microsphere size relationship was revealed. The effect of surface modification on the silica aerogel microspheres was also examined. 

## 2. Experimental

### 2.1. Materials

n-Heptane, n-hexane, Tween 80, Span 80, and ethanol with a purity of 99% were purchased from Fuyu Fine Chemical Co. (Tianjin, China). n-Butyl alcohol (≥99.5%) was purchased from Richjoint Chemical Reagent Co. (Shanghai, China). Nitric acid (HNO_3_, 65%), tetraethoxysilane (TEOS, 28%), trimethyl chlorosilane (TMCS, 99.9%), and ammonia hydroxide (NH_4_OH, 25%) were purchased from Guangzhou Chemical Reagent Co. (Guangzhou, China). Hexamethyldisiloxane (HMDSO, 99%) was purchased from Aladdin Chemical Reagent Co. (Shanghai, China). Alkali silica sol (30 wt % SiO_2_, density 1.20 g/cm^3^) was from Sui Xin Chemical Co. (Guangzhou, China). All chemicals were used as received without further purification.

Detailed information of the materials is shown in Supplementary Materials Table S1.

### 2.2. Preparation of SiO_2_ Aerogel Microspheres

**Figure 1:**
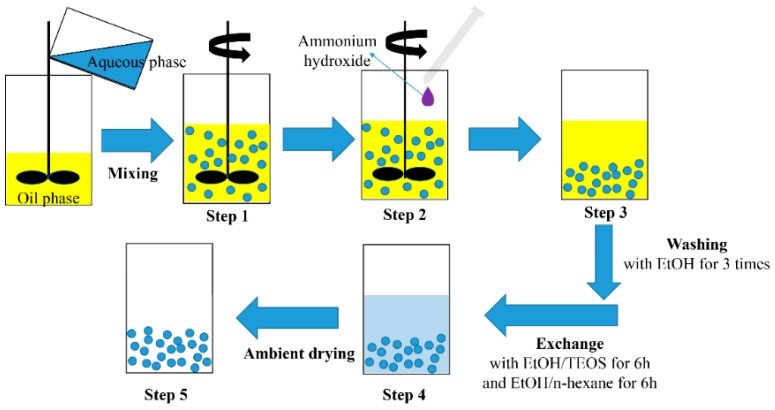
Flow chart showing the steps to synthesize the silica aerogel microspheres.

Overall, silica hydrogel was prepared by a two-step process, and silica hydrogel microspheres were synthesized via an emulsion method. Initially, 10 wt % nitric acid was mixed with 30 wt % alkali silica sol at 1:5 volume ratio, and the pH value of the solution reached approximately 2 after mixing. An aqueous phase was obtained by drop-wise addition of ethanol into the aforementioned mixture under constant agitation. The volume ratio of ethanol and alkali silica sol was 1.6:1. By using Span 80 and Tween 80 as composite emulsifier (10.3 mL) and n-butyl alcohol (3.0 mL) as a co-emulsifier, the resulting system was mixed with n-heptane (66.7 mL) under continuous agitation for 30 min at 30 °C to form an oil phase, which was subsequently mixed with the aqueous phase. The mixture was agitated for over 30 min until it was uniformly dispersed and the emulsion was obtained (Step 1 in Figure 1). A two-bladed axial stirrer (5 cm in diameter) was used to emulsify ca. 80 mL mixture in a 250 mL vessel (8 cm in internal diameter). 

Ammonia hydroxide aqueous solution (2.0 mol/L) was added drop by drop to the emulsion, changing the pH of the system to 8 (Step 2 in Figure 1). The system was agitated for another 20 min, during which silica hydrogel microspheres formed (Step 3 in Figure 1). After agitating, the container with wet gels was sealed, and the gels were aged at room temperature for 1–3 h. The prepared micron-sized silica hydrogel was washed three times after being soaked in an ethanol bath to remove the oil phase. After washing, the silica hydrogel was immersed in a container containing an ethanol/TEOS (3:1 volume ratio) solution for 6 h, and ethanol/n-hexane (3:1 volume ratio) solution for another 6 h (Step 4 in Figure 1). As the solvent exchange reaction proceeded, the water present in the hydrogel was displaced. The displaced water was then removed from the container, and the gel was dried by an oven under an ambient pressure at 80 °C for 6 h. The experimental procedures to synthesize the silica aerogel microspheres are shown in Figure 1. Control samples with surface modification were also prepared by immersing the silica hydrogel microspheres in a container containing HMDSO/TMCS/n-hexane (2:3:15 volume ratio) solution for 8 h and dried under an ambient pressure at 80 °C for 6 h.

It is highly desirable to understand how the dimension of the silica aerogel microspheres are affected by the processing parameters. As such, the following key parameters—including total concentration of the composite emulsifier, volume ratio of the aqueous phase and the oil phase, Span 80/Tween 80 mass ratio in the composite emulsifier, and agitation rate—are varied to study how such parameters will affect the size of the resultant silica aerogel microspheres. The study was performed by a step-by-step process and the purpose is to solve problems such as uneven filling or difficult recovery in the process of using aerogel powders. The identifications of the corresponding samples are summarized in Table 1.

### 2.3. Characterization

The microstructure of the silica aerogel microspheres was characterized using a field emission scanning electron microscope (Nova NanoSEM 430 m, FEI, Czech Republic, The Netherlands). The Fourier transform infrared spectroscopy (FTIR) spectra of the microspheres prepared by different formulations and processes were recorded using a spectrophotometer (Vector 33-MIR, Bruker Optic, Ettlingen, Germany) ranging from 500 to 4000 cm^−1^. For this analysis, aerogel microspheres were ground and mixed with KBr. 

The tapping density of the aerogel microspheres was obtained by filling them in a cylindrical column of known volume following with vibration for two minutes. Then the density was calculated from its mass to volume ratio. Each sample was tested seven times and the average value was reported as the tapping density of the aerogel microspheres.

The specific surface area and pore size distribution (PSD) were estimated using a surface area and porosity analyzer (NOVA4200e, Quantachrome Instruments, FL, USA). The aerogel microspheres were initially degassed at 300 °C for 3 h and the N_2_ adsorption-desorption isotherms were obtained at 77 K. The specific surface area was calculated using the Brunauer-Emmett-Teller (BET) method, whereas the pore size distribution was determined by the Barrett-Joyner-Halenda (BJH) method. The total pore volume was calculated at p/p_0_ = 0.99 G. When not corrected for the micropore volume, the BET-analysis of the isotherm yields a *C*-parameter that increases with an increasing microporosity, and aerogel skeleton changes volume during the measurements [21].

The hydrophobicity of the samples was analyzed by a Drop Shape Analysis System (OCA40 Micro, Dataphysics Co. Ltd., Regensburg, Germany) to determine the contact angle of the silica aerogel microspheres.

## 3. Results and Discussion

### 3.1. Formation Mechanism of Silica Aerogel Microspheres

**Figure 2:**
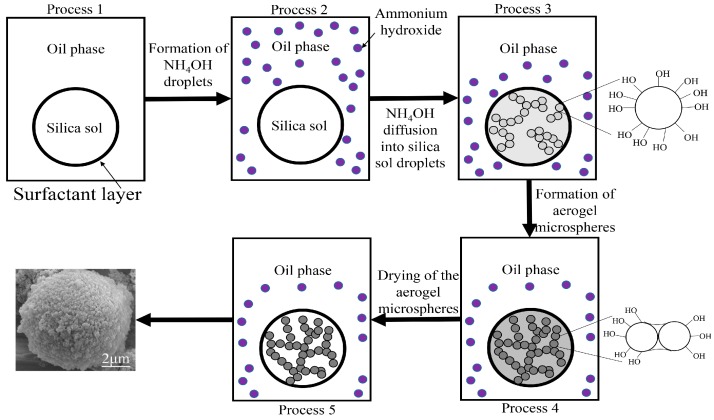
Proposed mechanism of the formation of silica aerogel microspheres.

Figure 2 shows the proposed mechanism for the formation of silica aerogel microspheres. When the silica sol is dispersed in the oil phase under constant agitation, sol droplets form in the oil phase and they are prevented from agglomerating by the surfactants. Only one droplet is shown in Figure 2 for the sake of clarity. The microspheres were formed through the following processes:

- Process 1: Formation of silica sol droplets upon its addition to the oil phase.
- Process 2: Formation of small droplets of NH_4_OH solution upon its addition to the emulsion containing silica sol droplets.
- Process 3: The NH_4_OH droplets surrounding the silica droplets and the condensation reaction starting at the surface of the silica sol droplets.
- Process 4: NH_4_OH diffusing into the silica droplets and resulting in its uniform distribution in the droplet (the formation of the wet gel).
- Process 5: Drying of the silica hydrogel microspheres, in order to remove the solvents in the pore of the gel.

### 3.2. Effect of the Processing Conditions on the Size and Morphology of the Silica Aerogel Microspheres

**Figure 3:**
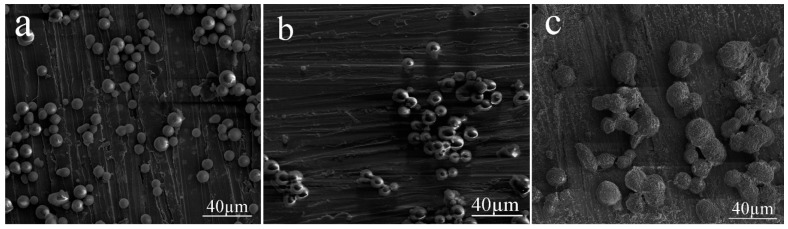
SEM images of the silica aerogel microspheres prepared at different emulsifier concentrations: (**a**) 0.30 g/mL; (**b**) 0.50 g/mL; and (**c**) 0.70 g/mL.

The effect of the concentration of the composite emulsifier on the size and morphology of the silica aerogel microspheres was investigated under the condition of a water/oil ratio of 0.3, an agitating rate of 400 rpm, and a mass ratio of Span 80/Tween 80 of 9:1. As one can observe from Figure 3a–c, there is no obvious change in the size of the silica aerogel microspheres. However, from Figure 3c one can observe that particles stuck together to form a chain-like structure and the particles became more significantly agglomerated, because it is more difficult to remove the emulsifier during the washing process. The result in Figure 3a showed that an emulsifier concentration of 0.30 g/mL was rather ideal for the synthesis of the silica aerogel microspheres in terms of minimum agglomeration. When the emulsifier concentration is even lower, there will be insufficient emulsifier attached to the interface and the emulsion will be unstable. As such, an emulsifier concentration of 0.30 g/mL was adopted for all of the following processing optimization. 

**Figure 4:**
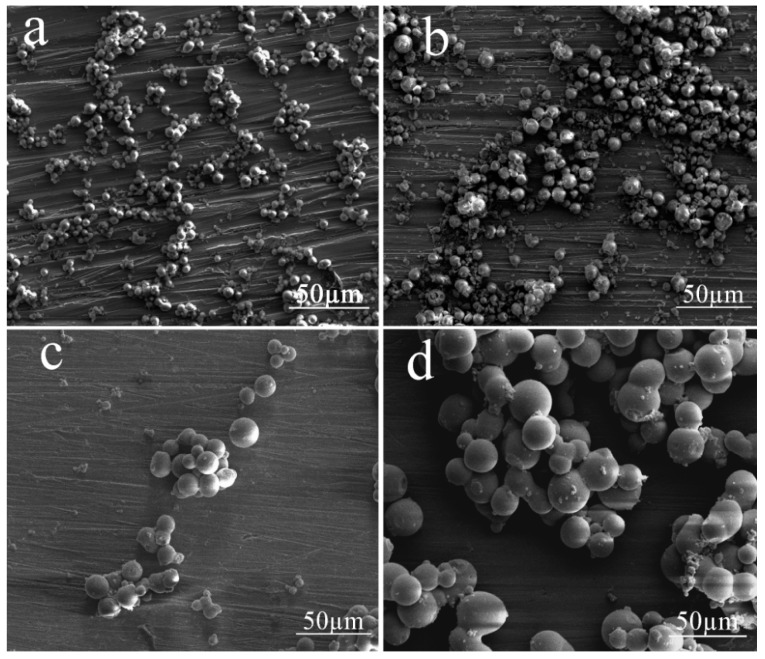
SEM images of the silica aerogel microspheres prepared at different water/oil ratios: (**a**) SA.V_0.1_; (**b**) SA.V_0.2_; (**c**) SA.V_0.3_; (**d**) SA.V_0.4_.

Figure 4 shows the influence of the volume ratio of the aqueous phase to the oil phase on the size and morphology of the resultant silica aerogel microspheres. In organic solvent, the size of the formed droplets is highly related to the water content in the oil phase. From the images in Figure 4, one can observe that the diameter of the silica aerogel microspheres increased with an increasing water/oil volume ratio. It is believed that because of the increase of water/oil ratio, the small droplets formed in the oil phase collided with each other, and the collision led to the merging of droplets to form larger ones, resulting in an increase of particle size and a broader particle size distribution (Supplementary Materials Figure S1). More than 400 microspheres were sized to determine the average particle size of these samples. Based on the results shown in Figure 4, an aqueous phase to oil phase ratio of 0.3:1 was adopted for the following explorations, as this ratio generated a relatively stable emulsion and a relatively high yield of silica aerogel microspheres.

**Figure 5:**
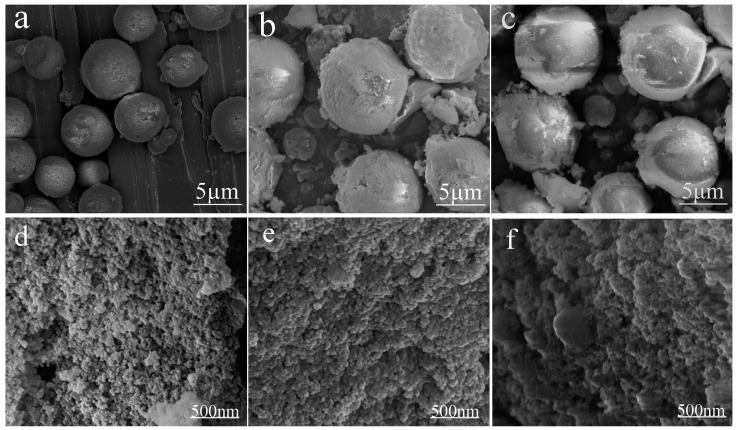
SEM images of the silica aerogel microspheres prepared at different compositions of the composite emulsifier: (**a**,**d**) SA.S_8_:T_2_; (**b**,**e**) SA.S_9_:T_1_; and (**c**,**f**) SA.S_9.8_:T_0.2_.

The composition of the composite emulsifier was also adjusted to examine how the composition would affect the size of the resultant silica aerogel microspheres. The diameter of the silica aerogel microspheres increased slightly when the mass ratio of Span 80 to Tween 80 changed from 8:2 to 9:1, but virtually no further size change occurred when the mass ratio of Span 80 to Tween 80 reached 9.8:0.2. The results, shown in Figure 5, suggest that the composite emulsifier composition only has a minor impact on the size of the synthesized microspheres. Figure 5d–f is the higher magnification images of Figure 5a–c respectively. It is obvious that porous microspheres formed based on clusters of particles. When the HLB value of the composite emulsifier is larger than 6, the emulsion becomes unstable, which is very undesirable for the formation of gel particles (Supplementary Materials Figure S2). In our case, the HLB value is 6.44 at 8:2 ratio and 5.37 at 9:1 ratio. For the consideration of a stable emulsion, a 9:1 ratio was chosen for most of our explorations. 

**Figure 6:**
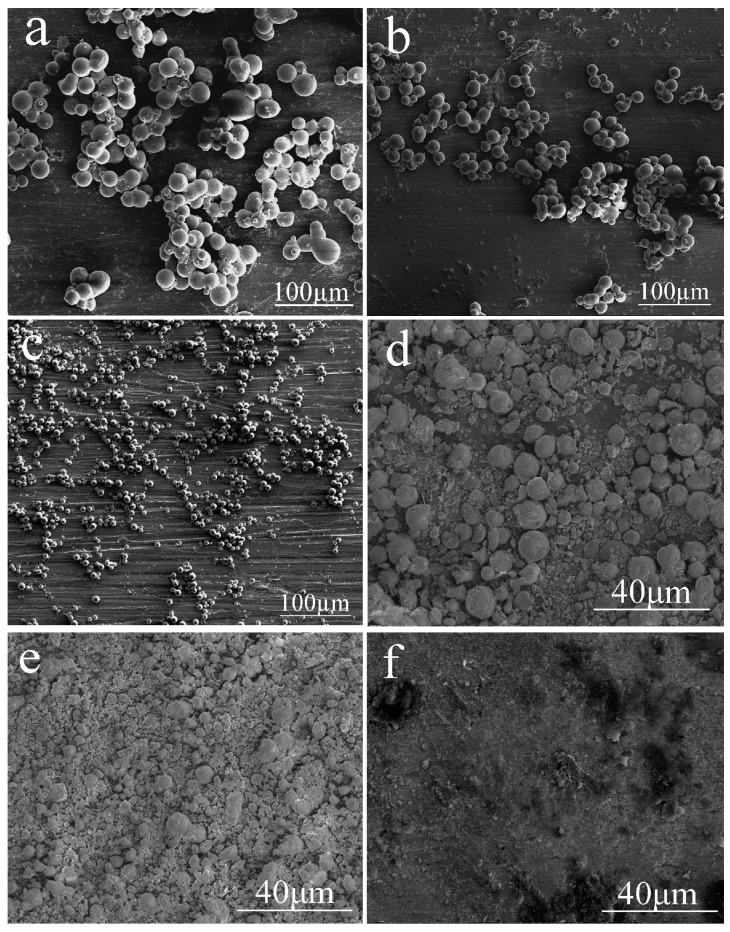
SEM images of the silica aerogel microspheres prepared at different agitating rates: (**a**) 300 rpm; (**b**) 400 rpm; (**c**) 500 rpm; (**d**) 600 rpm; (**e**) 700 rpm; and (**f**) 800 rpm.

In order to investigate the effect of the agitating rate, a range of 300 to 700 rpm were adopted. It can be seen from Figure 6 that the silica aerogel microspheres prepared at 300 rpm exhibit a relatively large mean size, ca. 18.8 µm (Figure 6a) and the diameter of the silica aerogel microspheres decreased as the agitating rate was increased to 400 rpm (13.6 µm, Figure 6b) and 500 rpm (6.8 µm, Figure 6c). When the agitating rate reached 600 rpm, the degree of fracture of the silica aerogel microspheres became significant (Figure 6d). The microspheres were almost completely ruptured when the agitating rate was increased to 700–800 rpm.

During synthesis, the silica sol was added to the oil phase under continuous agitation, and the formed tiny droplets were dispersed in the oil phase. The gel can maintain droplets form using ammonia to adjust the pH value of the emulsion. The high-speed agitation was effective to promote the spread of ammonia and form aqueous phase gel quickly.

Increasing the agitating rate results in a higher energy input to the system, which allows the building of a larger interfacial surface area, thus the dispersed droplets become smaller, and consequently the gel microspheres will have a smaller mean size [22]. However, when the shear stress reaches the threshold that can rupture the silica aerogel microspheres, a higher agitating rate will increase the degree of fragmentation of the aerogel microspheres.

These results suggest that agitation generates two effects: (1) disperse sol into smaller microspheres [23] and (2) possibly rupture the formed silica aerogel microspheres because of the high shear stress [24], and also show that agitating rate can be adopted to effectively tune the diameter of the silica aerogel microspheres. However, too high a shear stress is not desirable as it will lead to significant rupture of the spheres. In our case, silica aerogel microspheres can be prepared when the agitating rate ranges from 300 to 500 rpm, so we chose 400 rpm for our experiments.

In brief, the above results suggest that total concentration of the composite emulsifier, agitating rate, and water–oil ratio are the three most important factors that influence the diameter of the silica aerogel microspheres.

### 3.3. Surface Modification of the Silica Aerogel Microspheres

**Figure 7:**
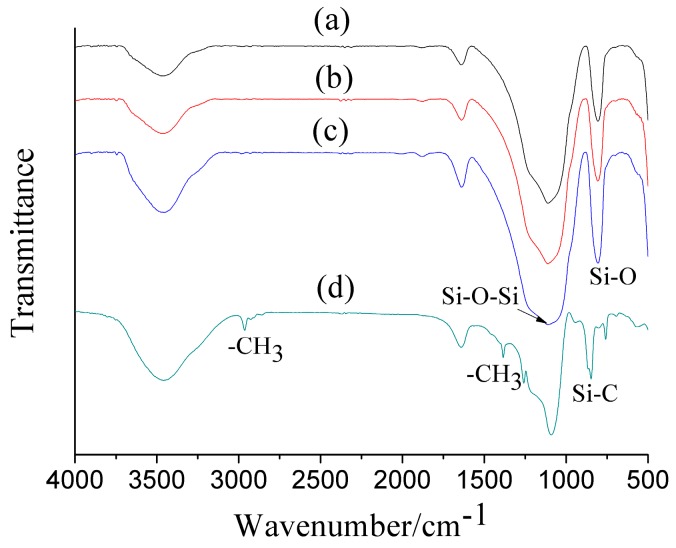
FTIR spectra of the unmodified silica aerogel microspheres. (**a**) SA.S_8_:T_2_; (**b**) SA.S_9_:T_1_; and (**c**) SA.S_9.8_:T_0.2_; and HMDSO/TMCS surface modified (**d**) SA.S_9_:T_1_(HT).

The FTIR characterization results of the surface modified and unmodified silica aerogel microspheres are shown in Figure 7 and summarized in Table 2. There were no obvious differences between the spectras of the unmodified silica aerogel microspheres (Figure 7a–c). The broad peak centered at ca. 3452 cm^−1^ is attributed to the O–H stretching band of hydrogen-bonded water [23]. The weak peak at ca. 1641 cm^−1^ is associated with the physically absorbed water [25]. The peak at ca. 810 cm^−1^ is related to the Si–O stretching vibration [26]. The peak at ca. 1112 cm^−1^ for Si–O–Si stretching vibration corresponds to the structure of the silica network [27]. However, the formation of Si–O–Si bonds collapse the pore network of the wet gel due to high capillary pressure [28]. Figure 7a–c shows the FTIR spectrum of the silica aerogel microspheres synthesized without surface modification, in which hydrogen bonded water (3452 cm^−1^) and physically absorbed H_2_O (1641 cm^−1^) peaks are present, but without Si–C peaks and –CH_3_ peaks, indicating that the unmodified silica aerogel microspheres are hydrophilic. 

Figure 7d shows the FTIR spectrum of the surface modified silica aerogel microspheres. The peaks at ca. 2963 and 1383 cm^−1^ are associated with the stretching vibration of methyl groups [26]. The peak at 844 cm^−1^ is due to Si–C bonding. These above peaks are attributed to the surface modification of the wet gels by HMDSO/TMCS [29]. Such a surface modification rendered the silica aerogel microspheres more hydrophobic than their un-modified counterparts (Supplementary Materials Figure S3).

### 3.4. Textural Properties of the Silica Aerogel Microspheres

**Figure 8:**
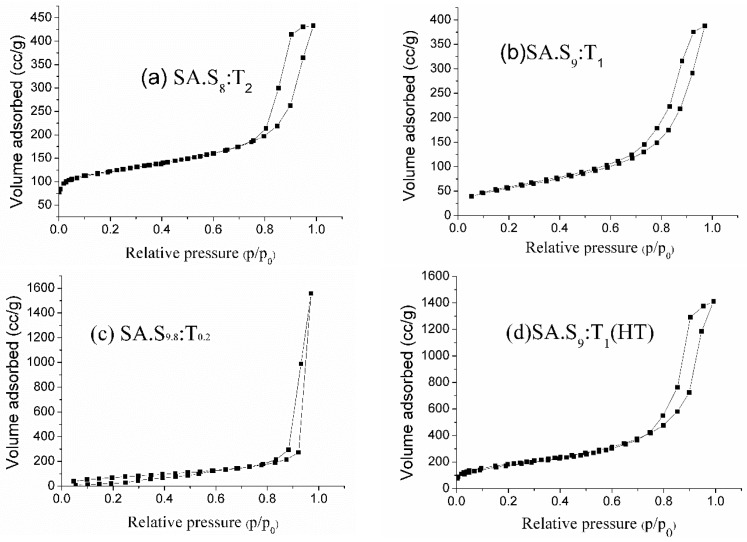
N_2_ adsorption-desorption isotherms of the silica aerogel microspheres obtained using different composite emulsifiers with and without surface modification.

The surface area and pore size distribution (PSD) of the silica aerogel microspheres were obtained by the standard BET and BJH methods, respectively. The nitrogen adsorption-desorption isotherms of the samples are shown in Figure 8 and their textural properties are summarized in Table 3.

The isotherm of samples SA.S_8_:T_2_, SA.S_9.8_:T_0.2_, SA.S_9_:T_1_, and SA.S_9_:T_1_(HT) are Type IV, which is a characteristic feature of mesoporous materials [30]. The desorption cycles of the isotherms showed a hysteresis loop which is generally attributed to the capillary condensation occurred in the mesopores. The shapes of the hysteresis loops are often identified with the specific pore structures. The adsorption-desorption branches of samples SA.S_9.8_:T_0.2_ is almost parallel and vertical over a range of p/p_0_ showing the H1 type of the hysteresis loop. Such a type of hysteresis loop is often associated with cylindrical pores, with interconnected networks [31]. The adsorption-desorption branches of samples SA.S_8_:T_2_, SA.S_9_:T_1_, and SA.S_9_:T_1_ (HT) showing the H_2_ type of the hysteresis loop, and correspond to pores with inkbottle shape [32].

The maximum amount of the N_2_ gas adsorbed by a porous solid obviously depends on the volume of the pores present in that materials. Sample SA.S_9_:T_1_ (HT) adsorbed a large amount of gas which saturated at 1411.5 cc/g at a relative pressure of 0.99 because of its relatively large pore volume (2.18 cm^3^/g).

Overall, as shown in Table 3, the silica aerogel microspheres modified with HMDSO/TMCS possess a higher surface area than the unmodified samples, because the gel network has a reduced irreversible shrinkage before APD caused by the surface silanol groups, and a more hydrophobic surface was achieved [33], which suggests that the modified sample may have a better performance than the unmodified ones in removing oil pollution in the water [34].

**Figure 9:**
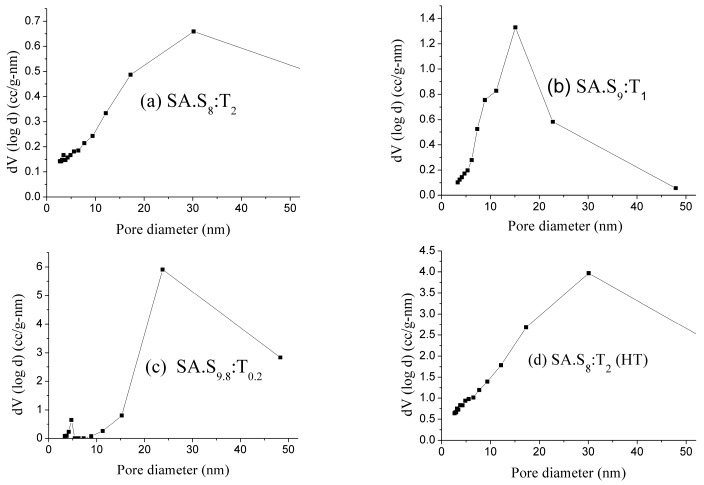
Pore size distribution profiles of the silica aerogel microspheres obtained using different composite emulsifiers with and without surface modification.

Figure 9 shows the pore size distribution of the synthesized silica aerogel microspheres using different composite emulsifiers. The pore size of all of the silica aerogel microspheres was in the mesoporous range (Table 3). We also can find that sample SA.S_9_:T_1_(HT) has a broader pore size distribution than others, which indicates that surface modification can reduce the collapse of the pore structure of the silica aerogel microspheres during the ambient pressure drying process.

## 4. Conclusions

Silica aerogel microspheres based on alkali silica sol were synthesized using the emulsion method. The effects of mechanical agitation speed, water/oil ratio, mass ratio of Span 80/Tween 80, and emulsifier content were investigated. The silica aerogel microspheres are mesoporous with an average pore diameter ranging from 6 to 35 nm depending on the processing conditions. The average diameter of the silica aerogel microspheres can be controlled within a range of 4–20 µm with an agitation speed of 300–500 rpm, a water/oil ratio of 0.1–0.3, and an emulsifier concentration of 0.30 g/ml. Samples modified with HMDSO/TMCS achieved a high specific surface together with a large pore volume. Such silica aerogel microspheres can be used to prepare large scale silica aerogels at an ambient pressure for widespread application.

## Figures and Tables

**Table 1:** Identification of the samples prepared under various conditions.

Series	Sample ID	Mass Ratio (Span 80:Tween 80)	Volume Ratio (Aqueous Phase:Oil Phase)	Vagitation (rpm)	Surface Modification Agent
**Volume Ratio Aqueous Phase: Oil Phase**					
SA.V_z_	SA.V_0.1_	9:1	0.1:1	400	None
	SA.V_0.2_	9:1	0.2:1	400	
	SA.V_0.3_	9:1	0.3:1	400	
	SA.V_0.4_	9:1	0.4:1	400	
**Mass Ratio Span 80:Tween 80**					
SA.S_x_:T_y_	SA.S_9.8_:T_0.2_	9.8:0.2	0.3:1	400	None
	SA.S_9_:T_1_	9:1	0.3:1	400	
	SA.S_8_:T_2_	8:2	0.3:1	400	
**Agitation Speed**					
SA.vAgitation	SA.v_300_	9:1	0.3:1	300	None
	SA.v_400_	9:1	0.3:1	400	
	SA.v_500_	9:1	0.3:1	500	
	SA.v_600_	9:1	0.3:1	600	
	SA.v_700_	9:1	0.3:1	700	
	SA.v_800_	9:1	0.3:1	800	
**Modified with HMDSO/TMCS**					
SA.S_x_:T_y_(HT)	SA.S_9_:T_1_(HT)	9:1	0.3:1	400	HMDSO/TMCS

**Table 2:** Summary of the FTIR characterization.

Wave	Vibration Mode	Group
3452 cm^−1^	Stretching vibration	–OH
2963 cm^−1^	Antisymmetric stretching vibration	–CH_3_
1641 cm^−1^	-	H_2_O
1383 cm^−1^	Stretching vibration	–CH_3_
1112 cm^−1^	Stretching vibration	Si–O–Si
844 cm^−1^	Antisymmetric stretching vibration	Si–C
810 cm^−1^	Stretching vibration	Si–O

**Table 3:** Textural properties of the silica aerogel microspheres obtained using different composite emulsifiers with and without surface modification.

Sample ID	Span 80:Tween 80 (wt:wt)	Tapping Density (g/cm^3^) *	BET Surface Area (m^2^/g) *	BET Measured Pore Volume (cm^3^/g)	Average Pore Diameter (nm)
SA.S_8_:T_2_	8:2	0.287 ± 0.001	451.2 ± 19.0	0.67	6
SA.S_9_:T_1_	9:1	0.195 ± 0.004	207.5 ± 5.0	0.61	12
SA.S_9.8_:T_0.2_	9.8:0.2	0.163 ± 0.008	273.7 ± 18.5	2.41	35
SA.S_9_:T_1_(HT)	9:1	0.112 ± 0.006	660.6 ± 4.4	2.18	13

* Including standard deviation.

## Supplementary Materials

The following are available online at www.mdpi.com/1996-1944/10/4/435/s1.
