# Supplementary material for: Preparation and Characterization of Silica Aerogel Microspheres
Source: Materials (Basel). 2017 Apr 20;10(4):435. doi: 10.3390/ma10040435 (PMC5506966; doi:10.3390/ma10040435)
Supplement: Supplementary file 1 [file materials-10-00435-s001.pdf]

Supplementary Information

Preparation and Characterization of Silica Aerogel Microspheres

Qifeng Chen, Hui Wang and Luyi Sun

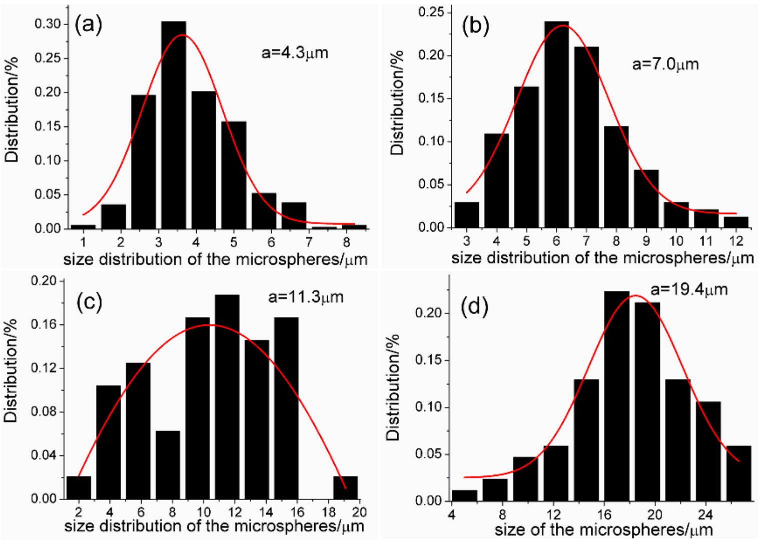

Figure S1. Particle size distribution of the silica aerogel microspheres prepared at different water/oil ratios: (a) SA.$V_{0.1}$, (b) SA.$V_{0.2}$, (c) SA.$V_{0.3}$, (d) SA.$V_{0.4}$.

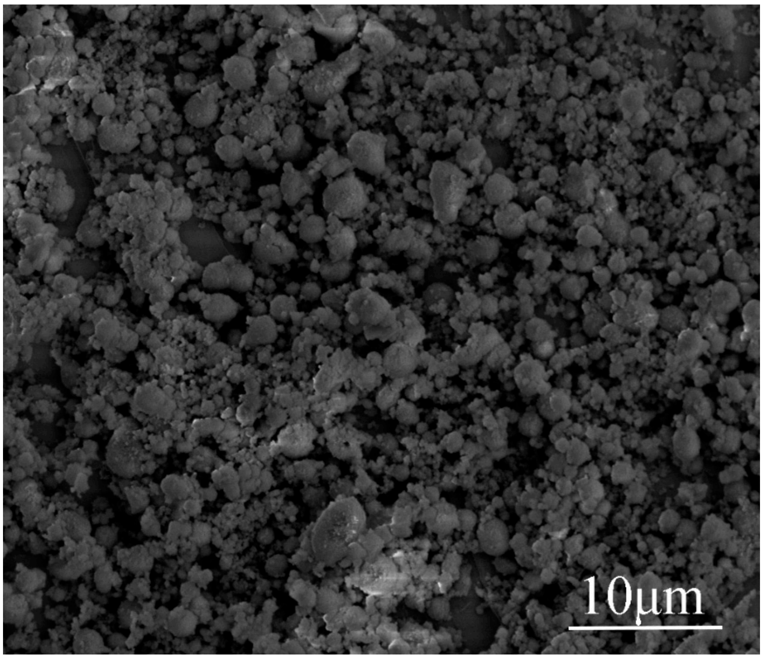

Figure S2. SEM image of the silica aerogel microspheres prepared at 65 wt% Span 80 and 35 wt% tween 80 (HLB = 8).

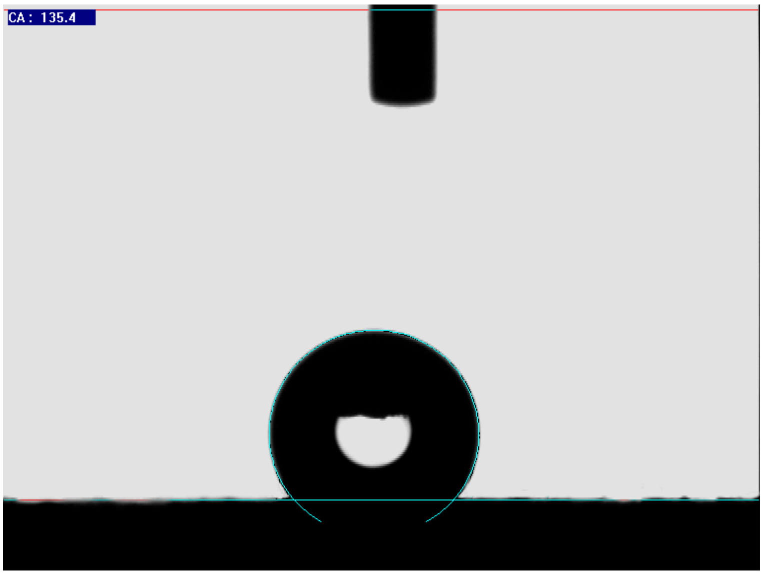

Figure S3. Contact angel of aerogel microspheres (SA.S_9:T_1(HT)).

Table S1. Detailed material information.

**Materials**	**Molecular Weight**	**Molecular Formula**	**Materials**	**Molecular Weight**	**Molecular Formula**
n-Heptane	100.20	C_7H_{16}	Tween 80	428.60	$C_{24}H_{44}O_6$
Span 80	428.60	$C_{24}H_{44}O_6$	Ethanol	46.07	C_2H_6O
n-Hexane	86.18	C_6H_{14}	n-Butyl alcohol	74.12	$CH_3(CH_2)_3OH$
Nitric acid	63.01	HNO_3	Tetraethoxysilane	208.33	$Si(OC_2H_5)_4$
Trimethyl chlorosilane	108.64	C_3H_9ClSi	Hexamethyldisiloxane	162.38	$C_6H_{18}OSi_2$
Ammonia hydroxide	35.05	NH_4OH	Alkaline silica sol	60.08	SiO_2
